# The effect of a low-carbohydrate diet on subcutaneous adipose tissue in females with lipedema

**DOI:** 10.3389/fnut.2024.1484612

**Published:** 2024-11-07

**Authors:** Julianne Lundanes, Mari Gårseth, Shannon Taylor, Rachelle Crescenzi, Michael Pridmore, Rune Wagnild, Åsne Ask Hyldmo, Catia Martins, Siren Nymo

**Affiliations:** ^1^Obesity Research Group, Department of Clinical and Molecular Medicine, Faculty of Medicine and Health Sciences, Norwegian University of Science and Technology (NTNU), Trondheim, Norway; ^2^Nord-Trøndelag Hospital Trust, Clinic of Surgery, Namsos Hospital, Namsos, Norway; ^3^Department of Diagnostic Imaging, Levanger Hospital, Nord-Trøndelag Hospital Trust, Levanger, Norway; ^4^Department of Radiology and Radiological Sciences, Vanderbilt University Medical Center, Nashville, TN, United States; ^5^Biomedical Engineering, Vanderbilt University, Nashville, TN, United States; ^6^ObeCe, Department of Surgery, St. Olavs University Hospital, Trondheim, Norway; ^7^Department of Nutrition Sciences, University of Alabama at Birmingham (UAB), Birmingham, AL, United States

**Keywords:** fat mass, body composition, magnetic resonance imaging, ketogenic diet, clinical trial

## Abstract

**Introduction:**

Lipedema is a common, yet underdiagnosed, subcutaneous adipose tissue (SAT) disorder. The main characteristics are SAT expansion in the lower extremities and arms, pain, and tenderness to palpation. It remains unknown if a low-carbohydrate diet (LCD) influences SAT in females with lipedema.

**Objectives:**

To evaluate the effect of a LCD low-energy diet, compared to a low-fat isoenergetic control diet, on calf subcutaneous adipose tissue area, muscle area, SAT/muscle ratio, calf circumference and body composition in females with lipedema.

**Subjects/methods:**

Adult females with obesity and lipedema were randomized to 1,200 kcal/day diets, either LCD or control (75 and 180 g/day of carbohydrates, respectively) for 8 weeks. Body composition was measured with bioelectrical impedance analysis, calf SAT area, muscle area, and circumference with magnetic resonance imaging and pain with brief pain inventory, before and after the intervention.

**Results:**

Thirteen participants were included (five in the LCD group), with a mean age of 46 ± 12 years and a BMI of 37 ± 6 kg/m^2^. A significant reduction in calf SAT area, calf circumference, and pain was observed in the LCD group only. Both LCD and control groups experienced a significant reduction body weight, fat mass, fat free mass, and muscle area, with no differences between groups. No significant changes over time were found for SAT/muscle ratio.

**Conclusion:**

A LCD has the potential to reduce SAT and pain in females with lipedema, despite a reduction in muscle mass in lipedema affected areas in both diet groups. Further studies are needed to confirm these findings and explore potential mechanisms.

**Clinical trial registration:**

NCT04632810: Effect of ketosis on pain and quality of life in patients with lipedema (Lipodiet). https://clinicaltrials.gov/study/NCT04632810.

## 1 Introduction

Lipedema is a fibrotic loose connective adipose tissue disease (1), characterized by a symmetrical increase of subcutaneous adipose tissue (SAT) in the lower extremities, pain, and tenderness to palpation in the affected areas (2). Lipedema affects almost exclusively females (1). Lipedema could be related to increased fluid and remodeling of connective tissue (1), which seem to occur alongside body composition changes driven by the large hormonal changes characteristic of puberty, pregnancy, and menopause states (3).

Given the lack of biomarkers for lipedema (4), diagnosis is based on visual inspection and review of medical history (5). However, recent evidence suggests that lower-extremity skin and SAT sodium content, as well as SAT area, measured with magnetic resonance imaging (MRI), are potential imaging biomarkers that can differentiate lipedema from obesity (6–8). This is of clinical relevance, as lipedema shares several features with obesity and is often misdiagnosed (3, 5).

The lipedema affected SAT differs from obesity related SAT (9), and has been claimed to be resistant to conventional dietary approaches (3, 5), due to fibrosis (1, 4). A low-carbohydrate (CHO) diet (LCD) has been proposed to relieve pain in lipedema patients, by damping inflammation (2), and/or by reducing total body water content due to glycogen depletion (10), which in combination with SAT reduction may decrease pressure on capillary nerves in the affected areas (11).

Despite the lack of well-designed studies investigating the impact of macronutrient composition of the diet on lipedema management, one case study (12), and a few intervention studies (13–16) using ketogenic (12), LCD (15, 16), or a mediterranean diet (14) report positive effects on lipedema symptoms (12), pain (12, 15, 16), body weight (12, 13, 16), circumference of calf, hip and waist (13, 15, 16) and fat mass (FM) in lower limbs (14). However, to our knowledge no study has compared the effect of diets with a different macronutrient composition on SAT in females with lipedema.

The primary objective of this secondary analysis was, therefore, to evaluate the effect of an eight-week LCD, compared to a low-fat isocaloric diet (control), on SAT area in the calf in females with lipedema. Secondary objectives were to evaluate and compare the effect of the two dietary interventions on muscle area, SAT/muscle ratio, calf circumference, body weight and composition.

## 2 Materials and methods

### 2.1 Study design

This study is a randomized controlled trial, where females with lipedema and obesity were randomized to either a LCD or a low-fat low-energy diet (LED) (control) for 8 weeks. Participants were randomized (1:1) to either arm using block randomization with stratification by body mass index (BMI) categories (30.0–34.9 kg/m^2^, 35.0–39.9 kg/m^2^, 40.0–44.9 kg/m^2^). Randomization was performed by a web-based randomization system developed and administered by the Faculty of Medicine and Health Sciences, Norwegian University of Science and Technology, Trondheim, Norway. This is a secondary analysis of a study aiming to investigate the effect of a LCD on pain and QoL in females with lipedema, and the main outcomes have already been published (17).

### 2.2 Participants

Females with lipedema, aged 18–75 years and a BMI between 30 and 45 kg/m^2^ were included in this study. The diagnosis of lipedema was performed by physiotherapists, while determination of lipedema type and stage was done by study personnel (2, 18). The participants had to be weight stable for the last 3 months (±3 kg). Exclusion criteria were acute and chronic kidney disease/failure, bariatric surgery, malignant disease, infectious disease, diabetes, psychological disorders, breastfeeding, pregnancy, current medication known to affect body weight, not mastering a Scandinavian language and enrollment in another obesity treatment program.

### 2.3 Dietary intervention

Both groups followed food-based 1,200 kcal/day diets for 8 weeks. The LCD had 75 g CHO (25 E%), 60 g protein (20 E%), 73 g fat (55 E%), while the control low-fat diet had 180 g CHO (60 E%), 60 g protein (20 E%), and 27 g fat (20 E%). The participants were advised to take a multivitamin (Nycoplus multi), and to drink 2 liters of non-caloric fluid every day and were asked to abstain from alcohol for the duration of the study.

### 2.4 Compliance

Participants were followed up by the study team weekly, either face-to-face or by phone. During these weekly follow-ups, participants were weighed, daily food records discussed, and potential side-effects recorded. Acetoacetate (AcAc) in urine was measured weekly using Ketostix (Bayer Corp, Elkhart, IN, USA), while blood ß-hydroxybutyrate (ß-HB) was measured at baseline, week 5 and week 9, using a ketone meter (Freestyle Precision Neo, Abbot, CA, USA). Participants who could not come to face-to-face meetings (particularly during COVID-19 pandemic) were asked to weigh themselves at home, provided with ketostix to measure AcAc in urine and followed up by phone.

The daily food records kept by the participants throughout the study period were analyzed for energy (kcal/day), and macronutrients (g/day) using a web-based analysis program for comparison of energy and nutritional content of food items (19) based on the Norwegian Food Composition Table (20).

### 2.5 Outcome variables

The following variables were collected at baseline and week 9.

#### 2.5.1 Magnetic resonance imaging

MRI was performed on the right calf, at the mid gastrocnemius muscle, using a 3 T-scanner (GE, Signa Architect). The exam consisted of 2-point Dixon imaging with the following parameters: 3D fast gradient echo, TR/TE = 27.768/6.618 ms, echo train length = 6, flip angle = 3 degrees, field-of-view = 192 × 192 mm^2^, matrix = 256 × 256, slice thickness = 5 mm, 18 slices, number of signal averages = 1, scan time = 3.57 min. Vendor-provided IDEAL Dixon algorithm calculated a separate series of fat- and water-weighted images for each slice. Regions of interest were segmented from fat- and water-weighted image contrasts including the SAT, muscle, and total leg. Segmentation was performed in an automated manner following published methods (6). Briefly, image intensity thresholding and morphological functions were used to automate the segmentation of SAT from fat-weighted images and muscle from water-weighted images. These regions were segmented from all 18 slices and the average values recorded for calf SAT area (mm^2^), muscle area (mm^2^), circumference (mm) and SAT/muscle (ratio) were quantified. The mean of the 18 slices from each participant were used in the analysis.

#### 2.5.2 Body weight, body composition and pain

Bioelectrical impedance analysis (BIA) (InBody720, Biospace CO., Ltd., Seoul, South Korea) was used to measure body weight, fat mass (FM) and fat free mass (FFM), both in kg and %, intracellular (ICW), extracellular (ECW), and total body water (TBW) in the fasting state. Pain was measured using the Brief Pain Inventory (BPI), with one question: “How much pain are you in right now?” (21, 22). The BPI assesses whole body pain intensity on a numeric rating scale, with 0 = no pain, and 10 = as bad as you can imagine (23).

### 2.6 Statistical analysis

Statistical analysis was performed using Stata 17 (StataCorp. 2021. Stata Statistical Software: Release 17. College Station, TX: StataCorp LLC.), and data presented as mean ± standard deviation (SD) unless otherwise specified. Histograms and Shapiro–Wilk test were used to assess the normality of the residuals. Statistical significance was set at *p* < 0.05. Group differences in the changes from baseline were estimated by linear mixed-effect models. The fixed part was specified in terms of two dummy variables; one for time and one for group differences (LCD vs. control diet) post intervention (w9), since the baseline means can be assumed to be the same, given the randomized nature of the study (24, 25). The mean difference in changes from BL is equivalent to the estimated mean group difference at w9. To account for within-subject correlations, a random intercept for subject was included. Independent sample t-tests were used to look at differences between groups in energy and macronutrient intake. Intention-to-treat analysis were performed. Figures were generated using GraphPad Prism (Version 10.0.2 for Windows, GraphPad Software, Boston, MA, USA).

## 3 Results

Baseline characteristics of the 13 participants (5 in the LCD and 8 in the control group) are presented in Table 1. The mean age was 46 ± 12 years, and BMI 37 ± 6 kg/m^2^.

**Table 1:** General characteristics of the participants at baseline.

	All participants (*n* = 13)	LCD (*n* = 5)	Control (*n* = 8)
Age, years	46.0 ± 11.5	50.2 ± 7.7	43.4 ± 13.1
Weight, kg	103.1 ± 18.8	95.3 ± 14.6	108.0 ± 20.3
Height, cm	166.5 ± 6.5	166.1 ± 4.6	166.8 ± 7.7
BMI, kg/m^2^	37.1 ± 6.3	34.5 ± 5.1	38.8 ± 6.7
Lipedema stage			
1, *n* (%)	4 (30.8%)	2 (40.0%)	2 (25.0%)
2, *n* (%)	8 (61.5%)	3 (60.0%)	5 (62.5%)
3, *n* (%)	1 (7.7%)	0 (0.0%)	1 (12.5%)
Lipedema type			
1, *n* (%)	2 (15.4%)	1 (20.0%)	1 (12.5%)
2, *n* (%)	2 (15.4%)	1 (20.0%)	1 (12.5%)
3, *n* (%)	2 (15.4%)	0 (0.0%)	2 (25.0%)
3 + 4, *n* (%)	7 (53.8%)	3 (60.0%)	4 (50.0%)

Data presented as mean ± SD. LCD, low-carbohydrate low-energy diet; Control, low-fat low-energy diet; BMI, body mass index.

### 3.1 Compliance

The participants’ daily energy and macronutrient intake, and ketone bodies (AcAc and BHB)’ concentrations is presented in Table 2. No differences in mean daily energy intake (EI) was seen between groups (1,181.8 ± 33.3 kcal vs. 1,176.1 ± 37.9 kcal, *p* = 0.821, in the LCD and control groups, respectively). The LCD group reported a lower daily CHO intake (66 ± 6 vs. 203 ± 17 g/day, *p* < 0.001), and a higher fat (76 ± 2 vs. 24 ± 3 g/day, *p* < 0.001), and protein intake (73.1 ± 6.3 vs. 51.8 ± 4.1 g/day, *p* < 0.001), compared with the control group.

**Table 2:** Mean daily energy and macronutrient intake and ketosis in both diet groups throughout the study.

	Group	Energy (Kcal/day)	CHO (g/day)	Fiber (g/day)	Protein (g/day)	Fat (g/day)	AcAc (mmol/L)	β-HB (mmol/L)
BL	LCD						0.0 ± 0.0	0.1 ± 0.1
	Control						0.0 ± 0.0	0.1 ± 0.1
W2	LCD	1,237.7 ± 91.3	72.8 ± 7.4*	26.3 ± 3.6	80.8 ± 12.3*	76.7 ± 6.8*	1.3 ± 1.6	
	Control	1,186.3 ± 33.5	205.9 ± 8.8*	28.1 ± 5.3	52.1 ± 8.1*	25.0 ± 2.9*	0.8 ± 1.4	
W3	LCD	1,182.0 ± 41.3	66.2 ± 7.0*	25.2 ± 3.6	76.8 ± 2.8*	70.3 ± 8.4*	2.6 ± 3.1*	
	Control	1,179.8 ± 54.7	204.2 ± 14.3*	27.8 ± 7.3	50.8 ± 7.7*	25.0 ± 3.4*	0.2 ± 0.3*	
W4	LCD	1,187.7 ± 25.4	66.3 ± 8.4*	25.2 ± 2.9	74.8 ± 8.4*	75.6 ± 5.4*	1.2 ± 0.8	
	Control	1,141.2 ± 122.0	199.3 ± 26.7*	26.9 ± 8.6	51.1 ± 5.7*	23.2 ± 6.0*	1.0 ± 1.4	
W5	LCD	1,184.5 ± 19.3	69.9 ± 10.5*	24.9 ± 3.8	70.0 ± 4.7*	77.2 ± 2.8*	0.9 ± 0.5	0.8 ± 0.3*
	Control	1,121.7 ± 226.9	195.3 ± 40.8*	24.9 ± 7.1	47.7 ± 9.2*	23.7 ± 6.7*	0.2 ± 0.5	0.1 ± 0.1*
W6	LCD	1,115.3 ± 92.3*	62.5 ± 10.5*	22.0 ± 2.9	68.7 ± 11.5*	73.5 ± 7.2*	2.9 ± 3.5*	
	Control	1,137.9 ± 143.9*	193.8 ± 40.5*	25.7 ± 7.4	50.5 ± 5.4*	22.6 ± 2.6*	0.1 ± 0.2*	
W7	LCD	1,172.4 ± 21.1	62.6 ± 6.8*	23.3 ± 4.2	70.4 ± 6.8*	77.3 ± 3.0*	2.0 ± 1.8	
	Control	1,215.0 ± 38.3	213.4 ± 10.9*	28.8 ± 6.5	53.6 ± 6.8*	24.8 ± 3.3*	0.0 ± 0.0	
W8	LCD	1,190.9 ± 101.1	63.9 ± 5.9*	22.5 ± 2.5	69.8 ± 10.1*	78.2 ± 8.8*	2.5 ± 1.0*	
	Control	1,214.1 ± 22.8	210.7 ± 15.2*	27.6 ± 7.4	52.9 ± 8.6*	25.0 ± 4.4*	0.0 ± 0.0*	
W9	LCD	1,183.6 ± 55.5	64.4 ± 6.2*	24.5 ± 3.6	73.6 ± 10.9*	76.9 ± 5.3*	1.9 ± 3.4*	0.6 ± 0.0*
	Control	1,212.8 ± 35.4	210.9 ± 14.3*	27.5 ± 7.8	52.1 ± 6.1*	25.3 ± 4.8*	0.1 ± 0.2*	0.3 ± 0.1*
Average	LCD	1,181.8 ± 33.3	66.1 ± 6.2*	24.2 ± 2.7	73.1 ± 6.3*	75.7 ± 1.6*		
	Control	1,176.1 ± 37.9	203.3 ± 17.3*	26.7 ± 6.4	24.0 ± 3.0*	24.2 ± 3.0*		

Data assessed by dietary registrations. Data presented as means ± SD. W, Week; CHO, Carbohydrates; LCD, low-carbohydrate diet; Control, low-fat low-energy diet; AcAc, Acetoacetate; β-HB, β-hydroxybutyrate. *Significant difference between groups. *p* < 0.05.

### 3.2 Magnetic resonance imaging

**Figure 1:**
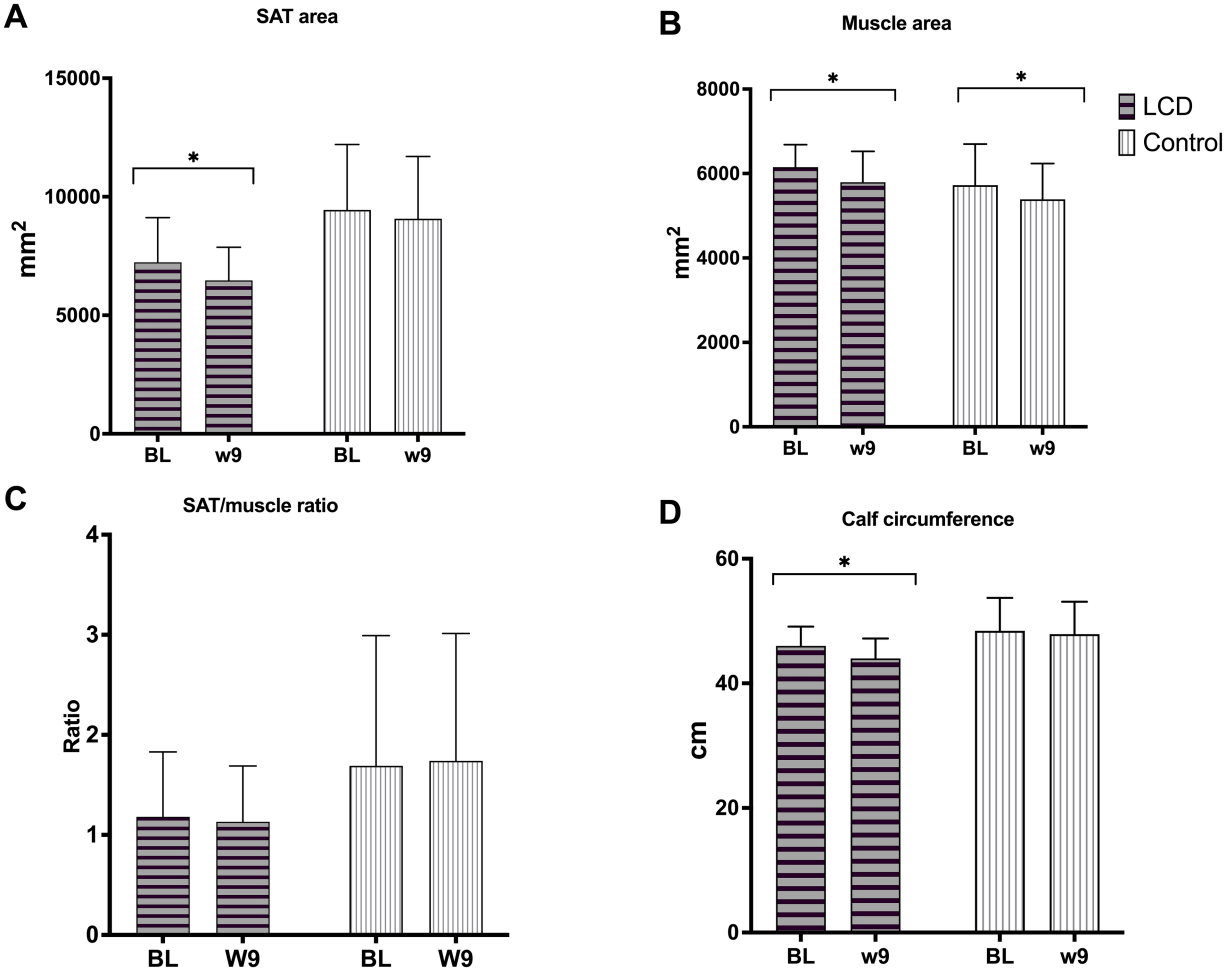
MRI metrics were assessed before (at baseline) and after dietary interventions (week 9) of low-carbohydrate and control diets, including **(A)** subcutaneous adipose tissue (SAT) area, **(B)** muscle area, **(C)** SAT/muscle ratio, and **(D)** calf circumference. Data are presented as mean + SD. *N* = 5 in LCD and *n* = 8 in control. LCD, low-carbohydrate diet; Control, low-fat low-energy diet; BL, Baseline; W9, week 9. *p < 0.05, significant changes within group from baseline to week 9.

Changes in SAT and muscle area, and calf circumference in both groups can be found in Figure 1 and Supplementary Table 1. A significant reduction in SAT area (−815.9 mm^2^, 95% CI: −1,423.0 to −207.0, *p* = 0.009) and calf circumference (−2.1 cm, 95% CI: −3.4 to −0.7, *p* = 0.002) was seen in the LCD group only. Both the LCD and control groups had a significant reduction in muscle area (−335.1 mm^2^, 95% CI: −620.1 to −50.2, *p* = 0.021; and −348.5 mm^2^, 95% CI: −574.9 to 122.2, *p* = 0.003, respectively). However, changes over time were not statistically significant different between groups.

### 3.3 Body weight, body composition and pain

**Figure 2:**
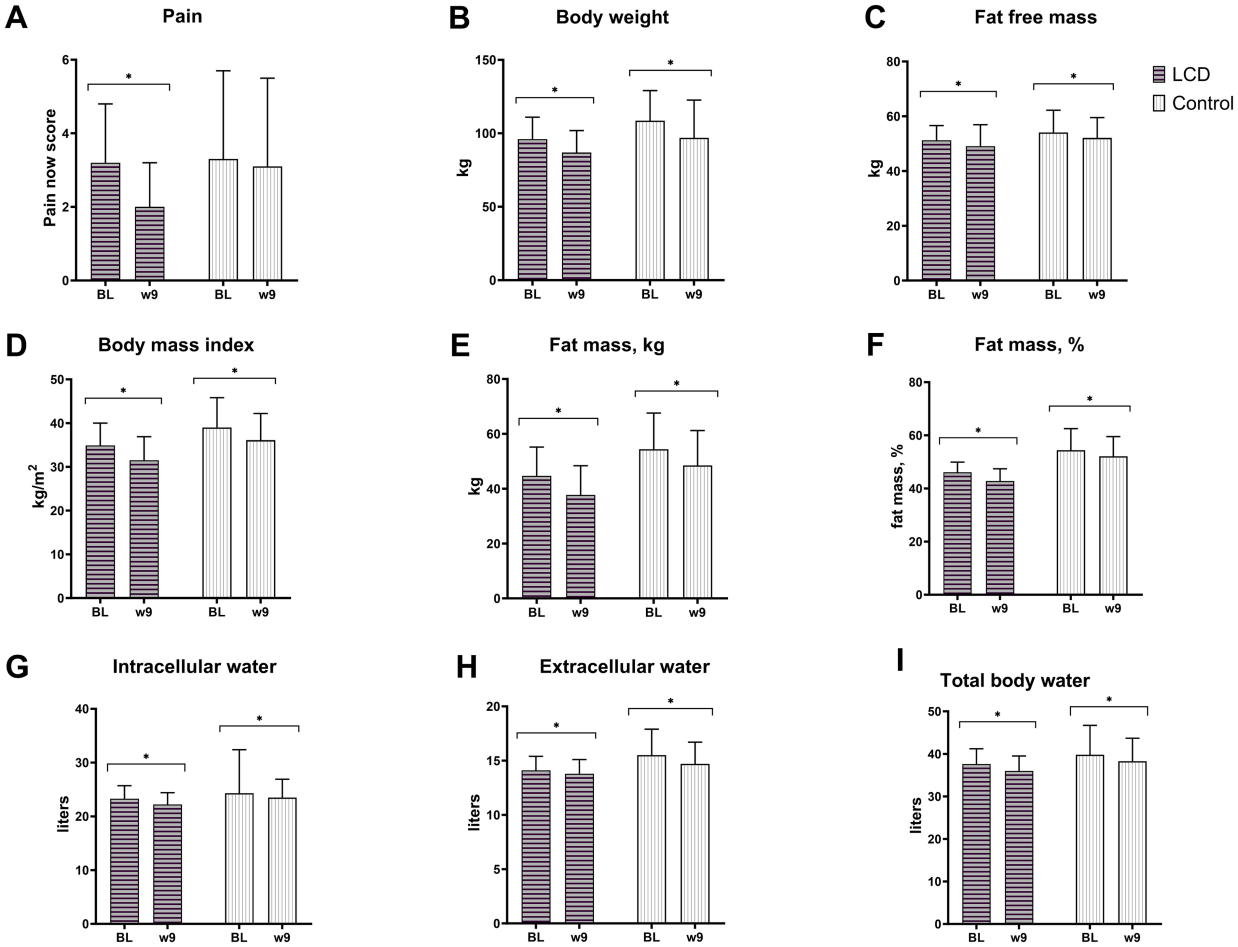
LCD and control groups at baseline and week 9 were assessed for **(A)** pain using brief pain inventory, **(B)** body weight, **(C)** fat free mass, **(D)** body mass index, **(E)** fat mass in kg, **(F)** fat mass in %, **(G)** intracellular water, **(H)** extracellular water, and **(I)** total body water using bioelectrical impedance. Data are presented as mean ± SD. *N* = 5 in LCD and *n* = 8 in control. BL, Baseline; W9, Week 9; LCD, low-carbohydrate low-energy diet; Control, low-fat low-energy diet. *p < 0.05, significant changes within groups from baseline to week 9.

Changes in body weight and composition, and pain in both groups can be seen in Figure 2 and Supplementary Table 2. Both groups had a reduction in body weight (−9.7 kg, 95% CI: −15.8 to −3.4 kg, *p* = 0.002 and −11.4 kg, 95% CI: −16.3 to −6.5 kg, *p* < 0.001, in the LCD and control groups, respectively), BMI, FM (both kg and %), FFM in kg, ICW, ECW and TBW. However, only the LCD group had a reduction in pain (−1.2, 95% CI: −2.3 to −0.1, *p* = 0.027). Changes over time for body weight, body composition, and pain were not statistically significant different between groups.

## 4 Discussion

The primary objective of this study was to evaluate the effect of an eight-week LCD on SAT area in the calf, compared to a low-fat isoenergetic diet (control) in females with lipedema. Secondary objectives were to evaluate the effect of the two diets on muscle area, SAT/muscle ratio, calf circumference, body weight and composition, and pain. Only the LCD group had a significant reduction in SAT area, calf circumference, and pain, but both groups experienced a reduction in body weight, FM, FFM, and muscle area.

Several hypotheses have been suggested to explain why a LCD may reduce SAT in the lipedema-affected areas, more than an isoenergetic low-fat diet. Keith et al. (11) proposed that the adipocytes in individuals with lipedema might exhibit impaired glucagon sensitivity, and/or insulin resistance. Adipocyte hypertrophy is promoted by hyperinsulinemia, and reducing CHO intake leads to decreased insulin secretion (26), potentially reducing adipocyte size (26, 27). The metabolic alterations induced by nutritional-induced ketosis could potentially lower insulin plasma concentrations sufficiently to facilitate lipolysis of lipedema adipocytes (11). This theory is reinforced by our findings of a significant decrease in SAT area and calf circumference only in the LCD group. Even though larger studies are needed to confirm these findings, our results show a promising role for LCD in reducing fat in the lipedema-affected areas.

The reduction in calf circumference measured by MRI in the LCD group in the present study is consistent with previous research (13, 15). Sørlie et al. investigated the effect of an eucaloric low-CHO high-fat (LCHF) diet for 6 weeks (15), and Jeziorek et al. the effect of an energy restricted LCHF diet compared to a medium-CHO medium-fat (MCMF) diet for 16 weeks (13), on calf circumference in patients with lipedema. Both studies reported a reduction in calf circumference following the LCD. Future studies should employ advanced MRI metrics of relaxometry to probe local tissue water content, given the impact of ketogenic diets on total body water.

A pain reduction of 1.2 on a numeric rating scale was found in the LCD group, which is in line with previous research (12, 15). Several mechanisms have been proposed to explain the reduction in pain in females with lipedema following a LCD. Specifically, reduced inflammation and less pressure on nerves due to water depletion and SAT reduction are possible mechanisms (11). There is increasing evidence for increased water content in the SAT in females with lipedema (6, 28). LCD result in water depletion (approximately 1.5–2.0 kg), due to glycogen breakdown (10, 29). However, in the present study similar reductions in ICW, ECW, and TBW were observed in both groups. It is possible that the reduction in pain in the LCD group in the present analysis was due, at least in part, to a reduction in calf SAT area in females with lipedema, as this was only observed in the LCD group. However, further research is needed, as the pain-relieving effect of a LCD is most likely multifactorial.

In the present study, both groups lost weight and FM, with no differences between them. In the study by Jeziorek et al. (13), a greater body weight and FM loss was seen in the LCHF (similar to our LCD diet) compared with the MCMF group after 16-weeks (13). However, EI differed between diets (1,677 kcal/day in LCHF group and 1,724 kcal/day in MCMF group, *p* = 0.001), which could account for some of the group differences, and the duration of the study was much longer than in the present study. Di Renzo et al. (14) also reported similar results after a 4-week modified Mediterranean diet, with a reduction in FM in legs and arms in females with lipedema, but no change in whole body FM (14). Participants also had a higher CHO intake (43.8 E%) compared to this present study (25 E%), and body composition was measured using dual-energy X-ray absorptiometry. Sørlie et al. found a significant weight loss, but no reduction in FM after an eucaloric 6-week LCHF diet (15). Contradicting results may be due to differences in EI, methods of body composition, and duration of the intervention.

A reduction in muscle mass from the MRI and FFM from BIA was seen in both diet groups in the present study. Loss of FFM is an undesired, but common side effect of diet-induced weight loss (30, 31). It has been suggested that a LCD diet could preserve or minimize the loss of FFM (32), also in females with lipedema (33). One study found FFM preservation after a 4-month very-low-energy ketogenic diet in 20 individuals with obesity (34). However, this was not the case in the present study, or other studies in individuals with overweight or obesity (35, 36). Exercise, degree of energy restriction, and rate of weight loss influence the proportional loss of FFM (37), and differences in these variables may account for discrepancy between studies. Resistance training and adequate protein intake may minimize the loss of FFM following energy restricted diets (32).

This study is a randomized controlled trial and from our knowledge the first of its kind to investigate the effect of different dietary interventions on calf SAT area in patients with lipedema using MRI. However, it has some limitations. First, the sample size is small, and the study was originally powered to investigate changes in pain. Second, the duration of the intervention was possibly too short to detect differences between groups in SAT changes over time. Third, even though the two diets were designed to be similar in protein, the LCD group had a higher protein intake compared to the control group, which may have affected the results. Additionally, a standard energy deficit was used for all participants, no physical activity data is available, and BIA is not a gold standard technique to assess body composition. Future studies should use dual energy X-ray absorptiometry to measure body composition changes over time, measure physical activity levels throughout the intervention, tailor energy deficit according to individual needs and consider type of carbohydrates, not only amount. Last, participants presented with different lipedema types (location of lipedema affected SAT), meaning that not all had lipedema SAT in the calf, which may also affect the present results.

## 5 Conclusion

A low-energy LCD has the potential to reduce calf SAT area and circumference, as well as pain in females with lipedema, despite a reduction in muscle mass in lipedema affected areas in both LCD and low-fat diet groups. Both diet groups had a reduction on whole-body FM, body weight, and BMI. The potential mechanisms behind SAT depleting in females with lipedema following a low-energy LCD should be explored in larger studies.

## Data Availability

The raw data supporting the conclusions of this article will be made available by the authors without undue reservation.
