# Supplementary material for: The effect of a low-carbohydrate diet on subcutaneous adipose tissue in females with lipedema
Source: Front Nutr. 2024 Nov 7;11:1484612. doi: 10.3389/fnut.2024.1484612 (PMC11578713; doi:10.3389/fnut.2024.1484612)
Supplement: Supplementary file 1 [file Table_1.DOCX]

| Supplementary table 1. SAT and muscle area, SAT/muscle ratio calf circumference in the two diet groups over time | | | | | | | | | | | | | |
| --- | --- | --- | --- | --- | --- | --- | --- | --- | --- | --- | --- | --- | --- |
|  | **Baseline** | | **Week 9** | | | **Difference within groups** | | | | **Difference between groups at w9** | | | |
|  | **Mean ± SD** | | **Mean ± SD** | | | **EMM** | **95% CI** | | **P value** | **EMM** | **95% CI** | | **P value** |
| SAT area, mm^2^ |  |  |  |  |  |  |  |  |  |  |  |  |  |
| LCD | 7233.8 ± 1885.5 | | 6470.6 ± 1394.4 | | | -815.0 | -1423.0 to -207.0 | | **0.009** | -477.5 | -1249.7 to 294.6 | | 0.225 |
| Control | 9440.2 ± 2 760.4 | | 9070.3 ± 2627.5 | | | -337.4 | -819.2 to 144.3 | | 0.170 |  |  |  |  |
| Muscle area, mm^2^ |  |  |  |  |  |  |  |  |  |  |  |  |  |
| LCD | 6150.5 ±533.7 | | 5794.1 ± 731.9 | | | -335.1 | -620.1 to -50.2 | | **0.021** | 13.4 | -346.9 to 373.7 | | 0.942 |
| Control | 5723.2 ± 973.7 | | 5387.9 ± 850.7 | | | -348.5 | -574.9 to -122.2 | | **0.003** |  |  |  |  |
| SAT/muscle ratio | | | | | | | | | | | | | |
| LCD | 1.2 ± 0.3 | | 1.1 ± 0.2 | | | -0.1 | -0.2 to 0.1 | | 0.312 | -0.1 | -0.3 to 0.0 | | 0.158 |
| Control | 1.7 ± 0.5 | | 1.7 ± 0.4 | | | 0.1 | -0.1 to 0.2 | | 0.326 |  |  |  |  |
| Calf circumference, cm | | | | | | | | | | | | | |
| LCD | 46.0 ± 3.1 | | 48.4 ± 5.3 | | | -2.1 | -3.4 to -0.7 | | **0.002** | -1.6 | -3.3 to 0.1 | | 0.062 |
| Control | 44.0 ± 3.2 | | 47.9 ± 5.2 | | | -0.5 | -1.5 to 0.6 | | 0.385 |  |  |  |  |
| Data presented as mean ± SD. Results from linear mixed model are presented as estimated marginal means with corresponding 95% confidence interval and p value. BL: Baseline. W9: week 9. LCD: Low-carbohydrate low-energy diet. Control: low-fat low-energy diet. SAT: subcutaneous adipose tissue. EMM: Estimated marginal means. CI: Confidence interval. | | | | | | | | | | | | | |
