# Supplementary material for: The effect of a low-carbohydrate diet on subcutaneous adipose tissue in females with lipedema
Source: Front Nutr. 2024 Nov 7;11:1484612. doi: 10.3389/fnut.2024.1484612 (PMC11578713; doi:10.3389/fnut.2024.1484612)
Supplement: Supplementary file 2 [file Table_2.DOCX]

| Supplementary Table 2. Body weight/composition and pain over time in the two diet groups | | | | | | | | | | | | | | |
| --- | --- | --- | --- | --- | --- | --- | --- | --- | --- | --- | --- | --- | --- | --- |
|  | **Baseline** | | | **Week 9** | | | **Difference within groups** | | | | **Difference between groups at W9** | | | |
|  | **Mean ± SD** | | | **Mean ± SD** | | | **EMM** | **95% CI** | | **P value** | **EMM** | **95% CI** | | **P value** |
| BMI, kg/m^2^ |  |  |  |  |  |  |  |  |  |  |  |  |  |  |
| LCD | 34.9 ± 5.1 | | | 31.5 ± 5.4 | | | -3.4 | -4.4 to -2.4 | | **<0.001** | -0.6 | -1.8 to 0.7 | | 0.376 |
| Control | 39.0 ± 6.8 | | | 36.1 ± 6.1 | | | -2.8 | -3.6 to -2.1 | | **<0.001** |  |  |  |  |
| Weight, kg |  |  |  |  |  |  |  |  |  |  |  |  |  |  |
| LCD | 96.0 ± 15.0 | | | 86.9 ± 15.0 | | | -9.6 | -15.8 to -3.4 | | **0.002** | 1.8 | -6.1 to 9.6 | | 0.656 |
| Control | 108.6 ± 20.5 | | | 96.9 ± 25.7 | | | -11.4 | -16.3 to -6.5 | | **<0.001** |  |  |  |  |
| Fat mass, kg | |  |  |  |  |  |  |  |  |  |  |  |  |  |
| LCD | 44.7 ± 10.5 | | | 37.7 ± 10.7 | | | -7.1 | -9.5 to -4.7 | | **<0.001** | -1.3 | -4.4 to 1.8 | | 0.405 |
| Control | 54.4 ± 13.2 | | | 48.5 ± 12.7 | | | -5.8 | -7.7 to -3.9 | | **<0.001** |  |  |  |  |
| Fat mass, % | |  |  |  |  |  |  |  |  |  |  |  |  |  |
| LCD | 46.1 ± 3.8 | | | 42.8 ± 4.6 | | | -4.1 | -6.7 to -1.6 | | **0.002** | -1.5 | -4.6 to 1.7 | | 0.365 |
| Control | 54.1 ± 8.1 | | | 52.1 ± 7.4 | | | -2.7 | -4.7 to -0.6 | | **0.011** |  |  |  |  |
| Fat free mass, kg | |  |  |  |  |  |  |  |  |  |  |  |  |  |
| LCD | 51.2 ± 5.4 | | | 49.1 ± 7.8 | | | -2.2 | -4.1 to -0.2 | | **0.029** | -0.3 | -2.7 to 2.2 | | 0.833 |
| Control | 54.1 ± 8.1 | | | 52.1 ± 7.4 | | | -1.9 | -3.4 to -0.4 | | **0.016** |  |  |  |  |
| Intracellular water, L | | | |  |  |  |  |  |  |  |  |  |  |  |
| LCD | 23.3 ± 2.4 | | | 22.2 ± 2.2 | | | -1.1 | -1.7 to -0.4 | | **0.002** | -0.3 | -1.2 to 0.6 | | 0.496 |
| Control | 24.3 ± 3.6 | | | 23.5 ± 3.4 | | | -0.8 | -1.3 to -0.2 | | **0.005** |  |  |  |  |
| Extracellular water, L | | |  |  |  |  |  |  |  |  |  |  |  |  |
| LCD | 14.4 ± 1.3 | | | 13.8 ± 1.3 | | | -0.6 | -1.1 to -0.1 | | **0.016** | 0.2 | -0.4 to 0.8 | | 0.554 |
| Control | 15.5 ± 2.4 | | | 14.7 ± 2.0 | | | -0.8 | -1.1 to -0.4 | | **<0.001** |  |  |  |  |
| Total body water, L | |  |  |  |  |  |  |  |  |  |  |  |  |  |
| LCD | 37.6 ± 3.6 | | | 36.0 ± 3.5 | | | -1.6 | -2.7 to -0.5 | | **0.004** | -0.1 | -1.5 to 1.3 | | 0.887 |
| Control | 39.8 ± 6.0 | | | 38.3 ± 5.4 | | | -1.5 | -2.4 to -0.6 | | **0.001** |  |  |  |  |
| Pain now |  |  |  |  |  |  |  |  |  |  |  |  |  |  |
| LCD | 3.2 ± 1.6 | | | 2.0 ± 1.2 | | | -1.2 | -2.3 to -0.1 | | **0.027** | -1.1 | -2.4 to 0.3 | | 0.112 |
| Control | 3.3 ± 2.4 | | | 3.1 ± 2.4 | | | -0.1 | -1.0 to 0.7 | | 0.782 |  |  |  |  |
| Data presented as mean ± SD. Results from linear mixed model are presented as estimated marginal means with corresponding 95% confidence interval and p value. BL: Baseline. W9: week 9. LCD: low-carbohydrate low-energy diet. Control: low-fat low-energy diet. EMM: Estimated marginal means. CI: Confidence interval. L: Liters. | | | | | | | | | | | | | | |
